# A Review of Chemosensation and Related Behavior in Aquatic Insects

**DOI:** 10.1673/031.011.6201

**Published:** 2011-05-17

**Authors:** José G. Crespo

**Affiliations:** Department of Biology, University of Utah, Salt Lake City, UT 84112

**Keywords:** antennal morphology, neurophysiology, odors, olfaction, sensilla

## Abstract

Insects that are secondarily adapted to aquatic environments are able to sense odors from a diverse array of sources. The antenna of these insects, as in all insects, is the main chemosensory structure and its input to the brain allows for integration of sensory information that ultimately ends in behavioral responses. Only a fraction of the aquatic insect orders have been studied with respect to their sensory biology and most of the work has centered either on the description of the different types of sensilla, or on the behavior of the insect as a whole. In this paper, the literature is exhaustively reviewed and ways in which antennal morphology, brain structure, and associated behavior can advance better understanding of the neurobiology involved in processing of chemosensory information are discussed. Moreover, the importance of studying such group of insects is stated, and at the same time it is shown that many interesting questions regarding olfactory processing can be addressed by looking into the changes that aquatic insects undergo when leaving their aquatic environment.

## Resumen

Los insectos que están adaptados secundariamente a ambientes acuáticos son capaces de percibir olores de una gran variedad de fuentes. La antena de estos insectos, como en todos los insectos, es la principal estructura quimiosensitiva cuyo aporte al cerebro permite la integración de la información sensorial que en última instancia termina en respuestas comportamentales. Solo unos pocos órdenes de insectos acuáticos han sido estudiados respecto a su biología sensorial y la mayor parte de los trabajos se han centrado en la descripción de los diferentes tipos de sensilias o en el comportamiento del insecto como un todo. En este trabajo se realiza una exhaustiva revisión de la literatura y se discuten las formas en que la morfología de las antenas, la estructura del cerebro y el comportamiento asociado a estos pueden promover una mejor comprensión de la neurobiología del procesamiento de la información quimiosensorial. Por otra parte, se establece la importancia del estudio de los insectos acuáticos y al mismo tiempo se demuestra que muchas preguntas interesantes sobre el procesamiento olfativo pueden abordarse estudiando los cambios que ocurren al abandonar el medio ambiente acuático.

## Introduction

Aquatic insects are an arbitrary group that includes insects that are associated with an aquatic or semiaquatic environment in one or more of their life stages (Daly 1984). Although only a small percentage (∼ 3%) of insects are aquatic, representatives are found in 13 insect orders (Daly 1984; Williams and Feltmate 1992) suggesting that partial aquatic lifestyle might be advantageous to a wide array of insects. Generally, aquatic insects are nymphs/larvae of terrestrial adults (see the section on Coleoptera below for an exception, Brown 1987) that spend some time in terrestrial environments during certain stage/s of their life cycle (see Plecoptera exception, Jewett 1963). These insects have been, in some cases, well studied because they are vectors of several diseases (e.g. Malaria, see Cook 1997), can be environmental quality biosensors (e.g. Kashian et al. 2007), and are utilized for understanding aquatic communities (e.g. Benke 1979; Waters 1979) and several other areas of ecology such as predator-prey interactions, competition, population dynamics, etc. (Resh and Rosenberg 1984).

According to the fossil record, aquatic insects appeared in the Triassic (Zherikhin in Belayeva et al. 2002), more than 150 MY after the appearance of insects (Gaunt and Miles 2002; Engel and Grimaldi 2004). This fact, along with the presence of a tracheal system in nearly all aquatic insects (Chapman 1998), supports the idea that these animals secondarily adapted to living in water (Resh and Solem 1984; Ross 1967; Pritchard et al. 1993). During the immature stages, insect behavior changes during development, which is interesting from an evolutionary point of view because there are several examples of species of different taxa that have secondarily adapted to living in water.

Insects that have adapted to aquatic habitats face a vast range of physical and chemical conditions that differ from those adapted to a terrestrial environment and thus, affect their physiology and behavior (Denny 1993; Vogel 1994). Obviously, sensory systems are also affected and since insects, like most invertebrates, rely on chemoreception as their main sensory modality (Hildebrand and Shepard 1997), this review will center on the current knowledge of their main chemoreceptor structures, i.e. their antennae, and chemosensory—associated behavior.

Chemoreception in aquatic insects is the perception of chemicals that originate in organic or inorganic sources, and if in aqueous solution, are perceived by gustatory sensilla or if airborne, perceived by olfactory sensilla. In aquatic insects, as in other aquatic animals, this distinction between taste and olfaction is vague, but is still used based on the structure, response, or particular location of the sensilla or the animal's behavioral response (Zacharuk 1980). As when released in air, chemical cues that propagate in water form a plume that in theory is well preserved at great distances from the source (Murlis *et al.* 1990). This, in addition to environmental conditions (e.g. turbidity of water, reduced light transmission, high habitat complexity, etc.), prompts aquatic insects to use chemical cues for foraging and in predator-prey interactions (Brönmark and Hansson 2000; Wisenden 2000).

The focus of this review is on the chemosensory adaptations of insects that live in an aquatic environment as nymphs/larvae before moving to a terrestrial setting as adults. Thus, the semiaquatic insects (e.g. *Leptysma marginicollis* [Order Orthoptera, Family Acrididae], *Pentacora signoreti* [Order Hemiptera, Family Saldidae], *Simyra sp.* [Order Lepidoptera, Family Noctuidae]), insects that live in interstices of the soil (e.g. *Hydraena sp.* [Order Coleoptera, Family Hydraenidae]), parasitoids of some aquatic insects (e.g. *Hydrophylita aquivolans* [Order Hymenoptera, Family Chalcidoidea]), and insects that live their whole lives in water (e.g. *Laccophilus sp.* [Order Coleoptera, Family Dytiscidae], and *Belostoma bakeri* [Order Hemiptera, Family Belostomatidae]) are not included. Of all the insect orders that have this dual way of life, only three hemimetabolous orders (Ephemeroptera, Odonata, and Plecoptera) and two holometabolous orders (Trichoptera and Diptera) have been studied so far.

In this review three aspects of chemosensation in insects are concentrated on: 1) the morphology of the antennae and its sensilla, which are the principal chemosensory organs; 2) the brain structures associated with processing chemical information (e.g. antennal lobes, mushroom bodies); and 3) the behavioral responses associated with chemical sensation (in particular olfaction). At the end of each section a short conclusion is provided on the current knowledge of each particular order. Finally, the importance of understanding how sensory information is encoded in the brain of these animals, how the data being collected will allow for better comprehension of aspects of neurobiology that remain uncertain, and the challenges that these animals face by switching from an aquatic to a terrestrial environment are discussed.

### Hemimetabolous orders

Three orders of hemimetabolous insects, i.e. insects that undergo an incomplete metamorphosis, have been studied regarding their sensory biology, namely Ephemeroptera, Odonata, and Plecoptera. These are described in the first part of this review.

### Ephemeroptera

#### Antennal morphology and types of sensilla Nymphs

A pair of filiform antennae, usually arising anterior or ventral to the eyes, are present in the nymphs. The scape and pedicel are usually well developed and the flagellum varies in the number of articles according to the species. For example, *Ephemera danica* has a flagellum with 26–27 segments (Rebora and Gaino 2008) while *Baetis rhodani* has 42–45 flagellar articles (Gaino and Rebora 1998). In this last species, a distal border of triangularshaped lobes is also characteristic of each antennal flagellar segment (Gaino and Rebora 1996). In general, the antennae have a great diversity of types of sensilla (Appendix 1), which are probably in concert with these animals' sensory requirements. The length of the antennae varies from short (or less than the head's width) to long (twice as long as the head's width; Edmunds et al. 1976). In the several species investigated so far, the following structures have been described: scolopidia, sensilla trichodea, chaetica, campaniformia, placodea, coeloconica, coeloconic-like, basiconica, and a new type of sensilla basiconica called “flat-tipped”. Although not all of the sensilla mentioned here, in the supplementary information and in following sections, have been reported to be chemosensory, they are included in this review because they are part of the antennal morphology. Furthermore, physiological data that show a non-chemosensory function (at least in the hemimetabolous orders) are not available.

#### Adults

After emergence from water, a radical modification of the nymph antennae occurs as the insect transforms into a subimago. In this stage, the winged mayfly is still not sexually mature and will ultimately molt to the reproductive stage (i.e. the imago stage). The small antennae of the subimago consist of a short scape, a well developed pedicel, and a thin filiform flagellum (Edmunds et al. 1976). The first two segments of the antennae are uniformly covered with microtrichia (Gaino and Rebora 1997, 1998) and the unsegmented flagellum presents cuticular ribs (Gaino and Rebora 1997). Although the subimago stage lasts for a short period of time and its antennae are not well developed, several authors have documented the presence of sensilla trichodea, coeloconica, campaniformia and basiconica (Appendix 1).

Ephemeroptera is the only order of insects that has two winged stages, i.e. a subimago and imago stage, even though some species do not molt into the imago stage (Peters and Peters 1977). The imago is the sexually mature stage and has antennae similar in form to the subimago. The antenna usually lacks sensilla in the scape and pedicel (e.g. Gaino and Rebora 1997, Gupta 1998, Slifer 1977) and cuticular scales replace the microtrichia in the scape and pedicel or only the pedicel of several species (Gaino and Rebora 1997, 1998). Gaino and Rebora (1997) suggested that these cuticular scales might indeed be squatiform sensilla, although they have not been described as such, and may perform a sensory function. These authors also reported that the unsegmented flagellum presents fanlike cuticular projections or a honeycomb-like structure.

In comparison to the nymphs, adults have less diversity of sensory structures in the antennae than would be expected from these animals' life style. Only three types of sensilla (i.e. trichodea, coeloconica, and campaniformia) have been described so far (Appendix 1).

#### Brain morphology

The brain morphology of Ephemeropteran nymphs has not been studied, but some information regarding the adults is available. Adults of Ephemeroptera, as well as Odonata, are considered to be primarily anosmic with respect to volatile odors because they lack the glomerular antennal lobes characteristic of Neoptera (Strausfeld et al. 1998). However, the absence of glomerular structures does not necessarily indicate (although strongly suggests) that olfaction is not a relevant sense for the animal (see Kristoffersen et al. 2008). With the present information, the suggested anosmic condition in Ephemeroptera is still debatable and studies on the electrophysiology of sensilla and detailed brain structure work are needed to resolve this issue.

Another important characteristic of the brain of these insects is the fact that the mushroom bodies present no evidence of a neopteran-like calyx (Strausfeld et al. 2009). Instead, a cluster of microglomeruli is localized in the most distal part of the pedunculus in the position where the calyx is normally located. Along with information from other phylogenetically relevant invertebrates, this observation led to the suggestion that the calyces receive olfactory input and that various sensory modalities reach the mushroom bodies indirectly through other protocerebral neuropils, not just olfactory ones (Strausfeld et al. 1998, 2009). Apparently, the role of the mushroom bodies in the palaeopteran insects would be that of integrating mechano- and optosensory rather than olfactory information, which might be correlated to the ancient environment in which they evolved (Strausfeld et al. 1998).

#### Behavior

##### Nymphs

Mayfly nymphs can be deposit-feeders, filter feeders, shredders, and/or scrapers that feed on detritus (e.g. leaf litter and associated microbioita), bacteria, diatoms, animal fragments (micro- or macroinvertebrates), and algae present on stone surfaces (Wiilliams and Feltmate 1992; Edmunds 1984). Besides gathering food, nymphs need to be able to avoid both invertebrate and vertebrate predators. Drift (i.e. the passive downstream transport of stream invertebrates of the benthos) has been repeatedly documented as a predator avoidance response (e.g. Corkum and Pointing 1979; Walton 1980; Corkum and Clifford 1980; Malmqvist and Sjöström 1987; Lancaster 1990; Flecker 1992; Culp and Scrimgeour 1993; Forrester 1994). The sensory mechanisms mediating drift and other avoidance behaviors (e.g. active swimming) when in the presence of predators (e.g. stoneflies, caddisflies, crustaceans, and fish) have been investigated on several occasions (see below). These studies have relied on static (e.g. freezing or showing tail curl behavior) and active (e.g. swimming, drifting, or crawling) behaviors to record mayflies' sensory capabilities when confronting an invertebrate or vertebrate predator.

#### Mayfly response to invertebrate predators

There are several invertebrate predators of mayfly nymphs. Stonefly nymphs are the most studied, but predatory mayflies and some crustaceans prey on mayfly nymphs as well. Several studies have attempted to demonstrate the nature of the predator cues that elicit the different behavioral responses in mayflies. For example, when measuring the number of nymphs in the region of highest stimulus concentration of an observation box, Peckarsky (1980) recorded that the number of individuals of certain species (e.g. *Ephemerella subvaria*), but not others (e.g. *Baetis phoebus*), decreased in the presence of chemical stimuli from a stonefly predator and later increased after the predator's removal. Furthermore, some nymphs (e.g. *Baetis bicaudatus*) were able to discriminate between predatory stoneflies (e.g. *Megarcys signata*) and a similar size omnivorous stonefly (*Pteronarcella badia*; Peckarsky and Dodson 1980) suggesting a chemical, tactile, or chemotactile mechanism of differentiation. Additionally, none of the mayfly species tested (seven species in total) reacted to the presence of the predators by visual cues alone indicating the importance of chemical information. These results were reinforced by the observations of Williams (1987), which showed that the same species studied by Peckarsky (1980) utilized a close-range (probably in the order of a few millimeters) chemodetection mechanism to sense the stonefly *Dinocras cephalotes.* The fact that only some species responded to predatory stonefly chemical cues was also found in the behavior of *B. rhodani* and *Rhithrogena nubile,* suggesting a species specific response to stonefly odors (Malmqvist 1992). In other cases, chemical cues sensed by mayflies have been shown to emanate from injured conspecifics (Huryn and Chivers 1999), which are supposed to be indirect cues of stonefly feeding, and even enhance the response to predator tactile stimuli, as in the case of *Paraleptophlebia adoptiva* (Ode and Wissinger 1993).

Alternatively, predator avoidance was also suggested to be a response to hydrodynamic cues. For example, Peckarsky (1987) and Peckarsky and Penton (1989a) suggested that *B. bicaudatus* utilizes its cerci as sensory structures in the presence of *Kogotus modestus* and that noncontact responses were probably due to pressure wave disturbances, i.e. hydrodynamic cues, created by the movement of the predator. Further evidence established that the mean predator-prey distance to elicit an evasive response was around 1–2 cm and the cue was again suggested to be hydrodynamic (Peckarsky 1996). However, in all these cases, chemical cues have not been discounted and recently the relevance of other arthropods' chemical cues, i.e. besides stoneflies chemical cues, in mayfly behavior was tested. Huryn and Chivers (1999) reported that *Siphlonurus mirus* reduced its levels of movement when exposed to chemicals from the predator mayfly, *Siphlonisca aerodromia*, and when *Stenonema* sp. was exposed to crayfish (*Orconectes rusticus*) conditioned water more mayflies displayed a “tail-curl” behavior, which is believed to serve as a mechanism of intimidation (Richmond and Lasenby 2006). Thus, hydrodynamic and chemical cues are probably both important for describing the mayfly response to invertebrate predators.

#### Mayfly response to vertebrate predators

Among the vertebrate predators of mayfly, fish (e.g. trout, sculpins, dace, and minnows) have been shown to greatly affect mayfly nymphs' behavior. Several studies have shown that mayflies use chemical cues to detect fish predators. Without rejecting other detection mechanisms, Kohler and McPeek (1989) suggested that the presence of chemicals from the mottled sculpin, *Cottus bairdi*, affected the feeding behavior of *Baetis tricaudatus.* Later on, it was found that, besides the effect of presence or absence of trout odor, mayflies adjust their behavior depending on experience (i.e. coming from a fishless or fish stream) and the time of the day (McIntosh and Peckarsky 1996). Similar to what was reported for stoneflies, these authors suggested that fish odor sensitized mayflies to the risk of predation. When the trout *Oncorhynchus mykiss* was added in a fishless stream, not only did *Baetis coelestis* reduce its daytime drift, but this behavioral response was also detected within 24 hours (Douglas et al. 1994) accounting for its persistence through time. Finally, the importance of the fish diet was demonstrated when *Salvelinus fontinalis* fed with *S. mirus* conspecifics, but not brine shrimp (a control), decreased the mayfly's movement activity (Huryn and Chivers 1999).

The fact that almost all the predator fishes utilized in these experiments fed mostly during the day (e.g. McIntosh and Toensend 1995) putting larger nymphs at higher risk than smaller ones, at least during this time of the day, persuaded researchers to look for behavioral differences among different mayfly sizes. Although, Huhta et al. (1999) found that large and small *B. rhodani* turn to nocturnal drift in the presence of minnow odor (*Phoxinus phoxinus*), size-related differences were recorded in natural environments by the addition of fish or its odor to a fish stream, i.e. a stream where a fish background odor is presumed to be present. A decrease in drift of large nymphs during the night and an increase in drift of small nymphs drift during day and night was the result of mayflies exposed to higher concentrations of fish odors, suggesting that mayflies discriminate between dissimilar concentrations of fish odor in natural environments (McIntosh et al. 1999) and that drift behavior changes depending on the developmental stage. Moreover, this behavioral response was observed within five minutes of exposure demonstrating how informative chemicals can be for these animals. However, other authors concluded that mayfly night drift probably occurred due to hydrodynamic, rather than chemical, cues from the predator (Culp et al. 1991; Tikkanen et al. 1994). Interestingly, some researchers have shown that brook trout odor can even induce morphological plasticity (e.g. develop longer caudal filaments) in mayflies, probably reducing predation rates on these insects (Dahl and Peckarsky 2002). These longer caudal filaments would improve predator detection, but have also been suggested to account for lower fitness and ultimately to a great reduction in mayfly biomass (Peckarsky et al. 2001; Peckarsky et al. 2002).

On the other hand, *B. rhodani* and *R. nubile* showed no behavioral changes to the predator *Cottus gobio* (Malmqvist 1992) and *Callibaetis ferrugineus* and seemed not to perceive brook trout odors (Caudill and Peckarsky 2003), suggesting again that evasive behaviors mediated by chemosensation may be species specific. In addition, the length of exposure to the stimulus is probably relevant. For example, Tikkanen et al. (1996) found that an immediate behavioral response in *B. rhodani* could be elicited only by actively foraging *P. phoxinus*, but not by its chemical cues alone or in combination with a fish model. However, when the fish model and chemicals were presented continuously (i.e. up to 17h.), an increase in the use of upper surfaces of tiles (where the food is located) peaked sharply in the first hours after dark. These results may indicate that mayflies use more than one type of cue to detect a predator and even invertebrate-vertebrate predator interactions cues (or maybe this interaction indirectly affects mayflies). Peckarsky and McIntosh (1998) studied the complex multiple-species interactions that occur between the mayfly, *B. bicaudatus*; the brook trout, *S. fontinalis*; and the nocturnal stonefly predator, *M. signata.* These authors concluded that both predators' odors reduced mature mayfly size and that while stoneflies increased night drift dispersal, trout suppressed feeding at night and drift. An interesting result was that fish odor changed the effect of stoneflies on *Baetis* drift in addition to reducing its drift directly, also indicating the importance of multiple prey-predator interactions (see also Soluk and Collins 1988).

As a final point, other types of odors (e.g. conspecific odors) alone or presented together with other types of stimuli have also been shown to elicit a behavioral response in mayflies. For example, Scrimgeour et al. (1994) studied the stimuli initiating changes in drift rates and position in substratum surfaces of three species of mayfly (*Ephemerella aurivilli, Paraleptophlebia heteronea,* and *B. tricaudatus*) and showed that the first two species responded to chemical stimuli alone, i.e. either predator or conspecific odors, and all three species responded to the hydrodynamic stimuli produced by the predator models, i.e. longnose dace and stonefly models, alone or in addition to chemical cues. This shows the importance of different types of stimuli on mayfly behavior when simultaneously presented. Furthermore, the behavioral response to chemical cues alone depended on the type of chemical stimulus in some species (e.g. *Ephemerella* responded to predator odors, but not to conspecifics odors) and was also species specific in others (e.g. *Paraleptophlebia* responded to conspecific odors, but *Baetis* and *Ephemerella* did not). The authors also showed that mayflies may be able to sense their own chemical stimuli, but do not respond unless other cues are also present (e.g. in the case of *Baetis*).

#### Adults

It is generally accepted that mayfly adults do not feed, although intake of water may occur (Takemon 1993), and they usually live for a very short period of time. Mating, oviposition and, in some cases, dispersal are the main functions of this stage. Unfortunately, the sensory mechanisms involved in these behaviors have been scarcely studied. Vision, by means of positive polarotaxis (e.g. Kriska et al. 2007), seems to be the predominant sensory modality involved in mating (Brink 1956; Brittain 1982) and oviposition (e.g. Kriska et al. 1998) in the majority of the species. However, McCafferty and Bloodgood (1989) described a distinctive copulating system and its associated reproductive structures in *Tortopus*, speculating that females could use a pheromone to attract males. Their speculation was also based on the observations that *Tortopus* and *Campsurus* mate at night and males have relatively small eyes. Mating attraction was further studied to find that, although first perception of females by males was visual, non-volatile chemical substances might be important after close physical contact (Landolt et al. 1997). Until now, the use of chemical signals for sex attraction has been speculative in Ephemeroptera and the antennae have not been the sensory structures suggested to be involved in it.

#### Conclusion

Ephemeropteran nymphs are equipped with chemoreceptors that allow them to, at least in some species, sense predators and injured conspecifics. On the other hand, adults apparently are anosmic (e.g. they lack a glomerular antennal lobe and mushroom bodies calyces) and all the current data indicate that they are visually driven animals. Thus, even though nymphs use chemical cues throughout their life stage, adults seem to be deprived of a chemical sense. These data and lack of chemoreception in adults begs us to ask *why* there is a loss of the chemical sense. Wouldn't it be advantageous for the adult to be able to select the best possible oviposition site (e.g. fish-free site) to ensure the success of its offspring? Or it may be difficult to find predation free areas, and so it would be more advantageous to spread the risk and oviposit in different locations.

### Odonata

#### Antennal morphology and types of sensilla Nymphs

The order Odonata is comprised of the suborders Zygoptera (Damselflies) and Anisoptera (Dragonflies). Species of both suborders have been the focus of several studies regarding the sensory biology of these insects and although the visual sense has been reported to be the primary sensory modality involved in prey detection and studied in detail (e.g. Sherk 1977), the nymphs of some species are less dependent on vision for prey capture. The antennae of the most phylogenetic basal forms of odonates are usually thick and show little differentiation between the base (scape and pedicel) and the apex (flagellum; Needham and Westfall 1955). In the most phylogenetically derived odonates, the antennae usually are seven jointed like in *Libellula depressa* (Gaino and Rebora 2001), but can also have fewer segments (e.g. *Epiophlebia superstes,* Faucheux 2007) and are of the filiform type (i.e. slender, cylindric, and greatly elongated). Sometimes the number of flagellomeres differs from one antenna to the other in the same individual and ornamentations can be present on the whole length of the antenna, as in *E. superstes* (Faucheux 2007). Besides the sensilla described in other parts of the body of the nymphs (e.g. Pritchard 1965b; Bassemir and Hansen 1980), sensilla trichodea, filliformia, basiconica, coeloconica, chaetica, campaniformia, and ampulliformia have been observed in the antennae (Appendix 2).

#### Adults

Even though adults are visual predators with exceptional vision (Sherk 1978) and the antennae of these animals undergo regressive development during the final molt (Needham and Westfall 1955), the antennal structures seem to be functional in the adult stage. In a comparative study of six species of zygopterans and 11 species of anisopterans, Slifer and Sekhon (1972) reported that the length of the antennae could be as short as 0.6 mm (in *Argia fumipennis,* Zygoptera) and as long as 2.1 mm (in *Anax junius,* Anisoptera); and they also reported that the flagellum of Zygoptera is strongly sculptured and undivided while the flagellum of Anisoptera is relatively smooth and composed of 2–5 segments. Among the sensilla described in several species are: ampulliformia, coeloconica, styloconica, and campaniformia (Appendix 2).

#### Brain morphology

Larvae have been almost neglected when it comes to the brain morphology and physiology associated with olfaction (for a review on other aspects of Odonata neurobiology see Mill 1982). Svidersky and Plotnikova (2004) describe the structural and functional organization of the mushroom bodies in last instar larvae, but they do so because the central nervous system is basically identical to that of the imago. Besides this report, only Plotnikova and Isavnina (2006) have studied the input of the antennal nerve to the brain in last instar nymphs of *Aeshna sp.* These authors found that the antennal nerve is connected to the lateral lobe of the protocerebrum and that the arborizations of such neurons are similar to those found in glomerular antennal tracts of *Musca domestica.* This result indicates that the lateral protocerebrum might at least be partly involved in the same type of processing that the antennal area is in other insects.

Adults are considered to be anosmic and they lack defined antennal lobes and calyces in the mushroom bodies. Nevertheless, the mushroom bodies are massive structures (Strausfeld 2009) and have been shown to receive afferents from the optic lobes (Svidersky and Plotnikova 2004). From the data available today, there seems to be little doubt about the poorly developed or actual existence of the olfactory system in adult odonates (but see Svidersky and Plotnikova 2006).

#### Behavior

##### Nymphs

Vision has been described as the most highly developed sense in many species of odonates, especially in visual predators like aeshnids and lestids, but others are tactile predators during the first nymphal instars or even throughout the whole larval development as in the case of *Calopteryx virgo* (Sherk 1977; Corbet 1999). The size and movement of the prey, in contrast to its shape, color, and odor (Tenebrio extract), have been observed to be important in stimulating feeding behavior (Pritchard 1965a). The use of mayfly and mayfly dummies (Rebora et al. 2004), other types of dummies (Etienne 1972), or immobilized tadpoles (Kanou and Shimozawa 1983) reinforced the idea that mechanical cues alone (Richards and Bull 1990) or mechanical and visual cues can elicit the release of the predatory labial strike. Although this mechanism has only been studied in Anisoptera, zygopterans presumably behave in a similar way.

As seen in Ephemeroptera, predator-prey interactions of odonates span a wide range (e.g. Caldwell et al. 1980) and in some lakes (usually in fishless lakes) these can be the top predators. However, odonates have been reported to have invertebrate (e.g. other odonates) and vertebrate (e.g. fish) predators in their natural habitats too (see below).

#### Dragonfly response to invertebrate predators

The main predators of odonatan nymphs are other Anisoptera, including larger conspecifics feeding on smaller ones (Corbet 1999), but other insects (e.g. aquatic heteroptera) also prey upon them. Predators can have important effects on mortality and growth of aquatic insects, including odonates (McPeek and Peckarsky 1998). Johansson (1993) showed that odonatan nymphs could detect and respond accordingly to the presence of an invertebrate predator. While the presence of large eyes might indicate visual stimulus to elicit an anti-predator response, several studies have shown that this is not the case. *Ischnura elegans* (Zygoptera) was able to detect the presence of the heteropteran predator *Notonecta glauca* in darkness by presumably using hydrodynamic or chemical cues, or even differentiate between this predator and a detritus feeding heteropteran (*Corixa punctata*; Heads 1985, 1986). Koperski (1997) found that the chemical cues of this same predator influenced prey consumption in *Enallagma cyathigerum* reinforcing the importance of chemical signals. When the behavior and hunting success of *E. cyathigerum* (Zygoptera) in the presence or absence of *Aeshna juncea* (Anisoptera) was studied, a marked response to visual and chemical cues from the predator was observed (Jeffries 1990). Also, *Pyrrhosoma nymphula* (Zygoptera) decreased its foraging activity when chemical stimuli alone and chemical and visual stimuli together of the predator *A. juncea* (Anisoptera) were provided (McBean et al. 2005). Furthermore, these authors demonstrated that predators fed with conspecifics significantly reduced their foraging activity, suggesting that this behavioral response occurs due to alarm pheromones released by conspecifics rather than by visual cues from the predator (Stoks 2001). This shows that vision is not the only important sense at least under these particular conditions. Hopper (2001) concluded that waterborne cues alone can cause *Pachydiplax longipennis* (Anisoptera) larvae to change their behavior in presence of different types of predators, and later on Mortensen and Richardson (2008) found that *Enallagma antennatum* (Zygoptera) foraging response is finely adjusted to predator/prey chemical signal combination (e.g. predator diet cues from *Tubifex sp.* and cues from injured *Tubifex sp.* elicit different responses). An even more interesting finding was that of the use of chemical and visual cues by small *Plathemis lydia* (Anisoptera) to detect larger cannibalistic conspecifics (Ferris and Rudolf 2007). However, these authors observed an opposite effect when compared to other studies, i.e. an increase in activity, spatial movement, and feeding behavior.

On the other hand, when *I. elegans* (Zygoptera) was presented with a caged anisopteran predator (*Anax imperator*) that allowed for chemical cues to be the main stimuli perceived, its foraging activity was not significantly reduced (Schaffner and Anholt 1998). Since a free-swimming predator did elicit a reduction in the feeding activity of *I. elegans,* the authors concluded that this response was probably due to visual cues. It is worth noting that this is the only paper that concluded that chemical cues are probably not involved and some authors have argued that the fact that certain animals do not perceive a high predator threat (like the presence of a caged predator) could explain the lack of behavioral response. For example, lamellae autotomy (i.e. the sacrifice of the lamellae to escape a predator) has been shown to influence escape behavior when nymphs were presented with fish kairomones (Gyssels and Stoks 2005, 2006). Thus, it may be the case that in addition to detecting the presence of a predator, these insects can evaluate the risk of being consumed according to the predator's spatial and temporal distribution. In addition, several authors also reported that the habitat background of odonates, i.e. coming from a fish or fishless lake, is a variable that has to be taken into consideration (e.g. McPeek 1990).

#### Dragonfly response to vertebrate predators

In lakes where fish are present, these can feed on several aquatic invertebrates, and odonatan nymphs have been reported to detect and avoid fish predators (Pierce 1988). Although this author suggested that probably visual and/or mechanical cues are the basis for predator detection, several later studies support a different type of predator detection mechanism in these animals. For one, *E. cyathigerum* responded to fish chemical stimuli by altering their feeding rate and diet composition (Koperski 1997). In other studies, the diet of the predator fish (i.e. if the fish was fed mealworms, damselflies, or fathead minnows) was shown to change the frequency of feeding bites, head bends, and moves in two *Enallagma* species (Chivers *et al.* 1996). Furthermore, the use of pike-naïve as well as pre-exposed to pike damselflies illustrated that nymphs can learn to identify predators through diet-related stimuli and that a single exposure to the chemical cues was enough to elicit the response (see also Wisenden et al. 1997).

Besides, as shown for ephemeropterans, the size of odonatan nymphs also seems to play a role in the response to fish (Dixon and Baker 1988) and even morphological changes (e.g. longer abdominal spines) have been observed to occur under predator selective pressure (Johansson and Samuelsson 1994; Johansson 2002; Johansson and Wahlström 2002; McCauley et al. 2008). For example, in *Leucorrhinia dubia* (Anisoptera), the growth of longer and wider abdominal spines due to the presence of perch waterborne chemicals (Arnqvist and Johansson 1998) seems to be an adaptation to life in a hostile environment. These spines may function as a defense mechanism or as a warning for predators, and demonstrate once more the importance of chemical signals in the lives of these insects.

#### Adults

Since newly emerged males are not sexually mature, they disperse before going back to the breeding sites. During this time adults feed until sexual maturity is attained. It is well known that adult odonates are visual predators and predominantly feed on flying insects according to their movement, size, and shape (Corbet 1980). It has also been established that at least some species of Anisoptera and Zygoptera possess a dorsal rim area in the compound eye that is sensitive to polarized light; indicating that orientation in their habitat and even flight directionality during migration could involve visual cues (Meyer and Labhart 1993). Besides vision, no reference to any other type of stimuli involved in hunting has been reported so far.

Mature males may defend their breeding territory to gain access to females and depending on the environmental and population conditions, both sexes have been shown to exhibit flexibility in their reproductive strategies (e.g. female passive choice in Irusta and Araújo 2007). Copulation can occur during flight or while resting and female acceptance may vary depending on male persistence to mate (e.g. Cordero and Andrés 2002). In addition to vision being the primary sense involved in mating, only a tactile recognition system involving the mesostigmal plates of the female and the inner surface of the male superior appendage during the tandem position seems to play a role (Robertson and Paterson 1982). No experimental data on the role of the antennae of adults in mating has been published so far, but due to their small size and lack of diversity and abundance of sensilla, chemical senses (at least involving the antennae) seem to be unimportant.

#### Conclusion

Odonates have well developed eyes, but being a primarily visual animal does not mean that other senses are not needed when exploring their environment. Nymphs have been shown to use infochemicals from a variety of predators and even learn to associate them with the presence of predators. In contrast, adults are considered to be anosmic (even though two types of antennal sensilla have been suggested to be chemosensory) and utilize vision for prey capturing and mating. Hence, once again it seems pertinent to ask what occurs with the chemical information that is so important in the immature stages, during the imago stage. Furthermore, if nymphs are able to learn, is this memory still present in the adult brain? As asked for Ephemeroptera, is the oviposition site decided according to the chemical information gathered during the immature stages? It is very interesting that both palaeopteran orders, i.e. Ephemeroptera and Odonata, have a chemosensory sensitive nymph but become anosmic as adults. The observation that these two palaeopteran orders drink water raises the possibility that water sampling might take place when choosing an oviposition site, implying that even though the adult loses the sense of smell, its sense of taste may be retained and employed during feeding and oviposition.

### Plecoptera

#### Antennal morphology and types of sensilla Nymphs

A pair of filliform antennae, formed by a scape, pedicel, and a long flagellum, is present in the nymphs. For example, in *Paragnetina media* the flagellum possesses 76–81 segments; and the first segment, called meriston, is the product of the fusion of several segments making it larger than the rest (Kapoor 1985). Plecopteran nymphs require a variety of sensilla to obtain information regarding temperature, mechanical, and chemical stimuli from the aquatic environment. Sensilla trichodea, basiconica, coeloconica, campaniformia, coniform complexes, and an unrecognized type of sensilla were observed in several plecopterans (Appendix 3).

#### Adults

The adults possess long fine antennae consisting of several segments that are covered with short setae (Williams and Feltmate 1992). As in the nymph, the adult antennae have similar shape and consist of a scape, pedicel, and a long flagellum. In *Allocapnia recta,* the 4 mm flagellum of the female is 1 mm longer than that of the male and, as seen in the nymph of *P. media,* its first segment is partially divided (Slifer 1979). This species also presents a different number of segments in the right and left antennae, and no differences were found between sexes. So far, in the species investigated, sensilla trichodea, chaetica, coeloconica, campaniformia, and an unrecognized type of sensilla have been found (Appendix 3).

#### Brain morphology

Until now no report regarding Plecoptera brain morphology or physiology has been published. Even though both nymphs and adults generally have well-developed antennae, no data are available on the structure of the antennal lobes or other parts of the brain of these animals.

#### Behavior

##### Nymphs

The diet of plecopteran nymphs varies depending on the species, developmental instar, or time of the day (Harper and Stewart 1984). Some species are herbivores-detritivores, others are omnivores-carnivores, and yet several change their feeding habit during development. Although many species are predators, other organisms (e.g. fish) predate on them too, and similar interactions to those reported for Ephemeroptera also occur. However, significantly less information is available regarding the importance of the different sensory modalities in predator-prey interactions and food searching.

Stoneflies have been reported to use their antennae to locate prey (e.g. Hynes 1941; Brinck 1949) and their efficiency depends on whether foraging occurs on surfaces more exposed to predators or not (Kovalak 1978). In the presence of predators, the nymphs' avoidance response may change their foraging behavior by confining them to a “safer” substratum (e.g. dark substratum; Feltmate and Williams 1989) where food may not be as abundant. Even if prey is abundant, the presence of a predator may increase the metabolic rate of the stonefly and reduce the efficiency of food assimilation as suggested by Duvall and Williams (1995). While the sensory cues involved in avoidance of fish predators have not been studied (except to a certain extent by Martinez 1987), some researchers have reported on the cues involved in food detection. The fact that some nymphs (e.g. Perlidae) have activity peaks during the crepuscule, or when very limited light is available (Hynes 1941; Brink 1949), is in accordance with the use of non-visual modalities to detect food. However, there are many animals that are adapted to see in low light intensity environments. When *K. modestus* and *M. signata* foraged with their eyes occluded or their antennae removed, Martinez (1987) showed that the antennae, but not the eyes, were necessary for feeding. This author also showed that chemical cues associated with competitors and prey elicited the appropriate response to each cue, stressing the importance of chemical information for these insects. On the other hand, detritivorous stoneflies, like *Pteronarcys pictetii,* were also shown to utilize chemical cues (in this case gustatory cues) when discriminating among food sources (Motyka et al. 1985).

In contrast to these findings, other authors found that mechanosensory cues were involved in the detection of prey items. The result that the stereotyped escape behavior of the ephemeropteran *Baetis*, but not that of *Heptageniids* or *Ephemerelids,* was attractive to stoneflies (Peckarsky and Penton 1989b) was shown to be the hydrodynamic stimulus *K. modestus* sensed when attacking its prey (Peckarsky and Wilcox 1989). Furthermore, these authors used a plastic model simulating the mayfly swimming wave patterns to test the importance of mechanosensory cues and at the same time eliminated any possible chemical or “normal” visual cues (it is important to note that these results contradict those of Martinez (1987) obtained with the same species). Two other stoneflies (*M. signata* and *D. cephalotes*) were shown to attack their ephemeropteran prey after antennal contact, suggesting that also mechanosensory stimuli initiate this behavior (Sjöström 1985; Peckarsky and Penton 1989a). So as in other examples presented above, different species seem to have evolved different detection mechanisms for their prey.

#### Adults

Although the antennae of adults are well developed, to my knowledge no research has been reported on the sensory biology of these structures. Reproduction of these animals has been reported to be primarily by vibrational communication (e.g. Stewart 1997; Virant-Doberlet and Cokl 2003; Sandberg and Stewart 2006), and regarding feeding habits very little is known. Many short-lived species do not feed (but do drink water) and many long-lived species feed on the green encrusting growth of bark, rotten wood (probably for the fungi component of it), or even honeydew; and in several species the intake of food is necessary to produce eggs (Hynes 1976). However, nothing is known about the sensory modalities used by the species that do feed as adults when searching for this food. It would be very surprising if those well-developed antennae had been retained without being advantageous for the insect's life.

#### Conclusion

In several species, the antennae of both nymphs and adults are conspicuous structures that, at least in nymphs, have been shown to be involved in feeding and predator-avoidance behaviors. Until now, no data have been collected regarding the importance of odor-mediated adult behaviors, brain morphology, or sensory physiology. Nevertheless, assuming that adults can perceive chemical Stimuli (based on the presence of several possible chemosensilla), what morphological changes take place in the antennae and sensilla of the nymph after developing into an adult? Are the same sensory neurons connected to the same brain structures in both stages? What kind of brain reorganization occurs in the adult and how does this restructuration affect the animal's biology? Although much more research is needed, especially in the imago, Plecoptera is the only order of the three hemimetabolous orders presented here that can possibly answer many of these questions because both the nymphal and adult instars have well developed antennae.

#### Holometabolous orders

Two orders of holometabolous insects, i.e. insects that undergo complete metamorphosis, have been studied regarding their sensory biology, namely Trichoptera and Diptera. As mentioned before, even though several studies have concentrated on aquatic Coleopterans, these insects are restricted to an aquatic life both as larvae and adults. Thus, these are not included in this review and only Trichoptera and Diptera will be covered in the second part of this review.

### Trichoptera

#### Antennal morphology and types of sensilla Larvae

In general the antennae of larvae of Trichoptera are not well developed and are represented by one or two apical papillae (Ross 1967). Denis (1984) reported that the basic structure of the antennae of several species was cylindrical with a large and rounded lobe, minute warts, and two tubercles in the middle. While in *Nectopsyche albida* the antenna consists of a short scape, long pedicel and a highly reduced flagellum (Tozer 1982), in *Melampophylax mucoreus* this has been described as a small peg positioned at the dorsolateral frontal edge of the head near the insertion of the mandibles (Spanhoff et al. 2005). In this last species, the tip of the single segmented antenna is laterally canted off and presents a plate structure, which could be interpreted as a multiporous plate sensillum by comparison to a similar structure in the larva of *Homoeosoma nebulella* (Lepidoptera: Pyralidae; Faucheux 1995).

Although other types of sensilla (e.g. basiconica and styloconica) have been described in the mouthparts of the larvae (Motyka et al. 1985; Spanhoff et al. 2005), only trichodea seem to be present on the antennae. Denis (1984) observed a single trichoid seta between the two tubercles on the medial side of the antennae of several species (except for *Goeridae pilosa*) and Tozer (1982) observed a similar structure in the apical portion of each antenna of *N. albida.* This last author suggested that the trichoid sensillum might be a mechanosensillum useful in locomotion and feeding activities as in Lepidopteran larvae. Thus, Trichopteran larvae seem to be deprived of antennal chemosensilla.

#### Adults

Quite opposite to what happens in the larvae, the antennae of the adult are very conspicuous and may vary in length according to the species and sex. For example, in *N. albida* the length of the whole antenna (i.e. the bulbous scape, relatively short pedicel, and long filiform flagellum) is about 32 mm in males and 16 mm in females (Tozer 1982), while in *Frenesia missa* the flagellum (probably the first two segments are very short) varied from 7–10 mm in both sexes (Slifer and Sekhon 1971). In the first species, sensilla are present on both sides of the flagellum and sexual dimorphism is also apparent in the size and shape of the scales, the presence of annulated sensilla in the male, and in the number of flagellar subdivisions (Tozer 1982). On the other hand, *F. missa* does not present antennal sexual dimorphism (but see sensilla description in Appendix 4) and the thread-like flagellum is divided into 45–50 subsegments in both sexes (Slifer and Sekhon 1971). In this last species, the flagellar subsegments are covered with microtrichia and large, flattened, sharp-tipped hairs (probably similar to the scales seen in Lepidoptera) are the most conspicuous structures. The following are the type of sensilla described in these two species (since two different types of classification were used by different authors, some of these categories could be actually the same): chaetica, campaniformia, squamiformia, thick-walled chemoreceptor pegs, thin-walled chemoreceptor pegs, thin-walled pegs in a depression, and a special type of thin-walled chemoreceptor called plate organs.

#### Brain morphology

Even though there is a close phylogenetic relationship between Trichoptera and Lepidoptera (Grimaldi and Engel 2005), and morphological similarities are known to exist, no information about the Trichoptera brain structure (e.g. antennal lobes, mushroom bodies, etc.) is available. Only two species of adult Trichoptera were mentioned in a paper on the phylogeny of a serotonin neuron in the antennal lobes of several insect orders (Dacks et al. 2006).

The extensive knowledge on Lepidoptera brain morphology and function and the available electrophysiological and histological techniques will most certainly prove to be very useful in future research in Trichoptera because of presumed similarities among these two groups. This will most definitely serve as a basis for comparison.

#### Behavior

##### Larvae

These insects are probably best known for the attractive caddis that some larvae build as shelters. Most trichopteran larvae feed on plant materials (although some are predaceous) and even though they are not very selective, they are greatly specialized for food acquisition (Wiggins 1984). Shredders have been observed to feed more heavily on leaves that are microbially colonized than on uncolonized ones, leading Motyka et al. (1985) to test for the response of larvae of *Pycnopsyche guttifer* (note that he also used plecopteran larvae of *P. pictetii*) to noncontact chemical compounds released by hickory and ash leaves. This species seemed to prefer colonized leaves after contact was already established indicating that prolonged arrestment on the chosen food might be triggered by gustatory cues. Later on Spanhoff et al. (2005) tested whether the antennae of *M. mucoreus* were involved in long-range food finding. Their results not only demonstrate that larvae with amputated antennae behave the same as those with intact antennae, but also suggest that contact chemoreception for identification of food patches may be achieved by sensilla in the maxillary palps and galea. Regarding predaceous larvae, experiments with *Plectrocnemia conspersa* show that vibrations of their irregular catching net (used to trap invertebrates) are transmitted to the larva and depending on the frequency, elicit feeding behavior (Tachet 1977).

Predator avoidance responses have not been studied so far. However, it may be the case that since a caddis protects some of the trichopteran species, these did not evolve a kairomone-mediated predator detection mechanism (suggested by Tachet 1977) as seen in other aquatic larvae.

#### Adults

Feeding habits in adult Trichoptera have been overlooked mainly because of the belief that they do not feed; but some species have been seen to feed on plant nectar (Crichton 1957) and functional mouthparts modified for sucking have been reported in six species (representing four families; Frings and Frings 1956). In contrast, Trichoptera mating behavior has received much more attention. In the search for exocrine glands that could secrete sex pheromones, Roemhild (1980) found secretory glands in the head and thorax of nine species of microcaddisflies (Hydroptilidae). Since these glands were observed only in males at the sexually active stage, these glands were suggested to be sex-pheromone production structures. However, Solem (1985), without finding the actual glands, demonstrated that the fourth abdominal sternite of *Rhyacophila nubila* was attractive to males. Exocrine glands were found to be associated with this abdominal sternite (Löfstedt et al. 1994), and Resh and Wood (1985) reported the presence of paired glands in the fifth abdominal sternite of *Dicosmoecus gilvipes* and two *Gumaga* species. The presence of exocrine glands was also found in *Hydropsyche angustipennis, Rhyacophila fasciata* (Löfstedt *et al.* 1994), *Molanna angustata* (Löfstedt *et al.* 2008), and in half of the 26 Trichoptera examined by Nielsen (1980). Interestingly, in all the species studied both females and males had a homologous gland system but its secretion was shown to be, at least in some cases, sex specific (e.g. Ansteeg and Dettner 1991). This suggests that these glands may produce compounds that have diverse roles (e.g. sexual in females and aggregational in males; Valeur et al. 1990 in Ansteeg and Dettner 1991).

Some authors suggested that caddisfly glands secrete defensive compounds against invertebrate predators (Duffield et al. 1977; Duffield 1981) and Ansteeg and Dettner (1991) found that some of these compounds had a very high toxicity for ants. Nevertheless, after Wood and Resh (1984) first demonstrated a chemically mediated sexual communication system in *Gumaga griseola,* many other reports showing similar results followed (e.g. Resh et al. 1987; Solem and Petersson 1987), suggesting a widespread use of this form of communication among sexes (see Ivanov 1993 for other basic communication signals between sexes). The timing of mate attraction and flight activity found in some species also reinforced these observations (e.g. Jackson and Resh 1991). Further confirmation came from electrophysiological experiments with identified compounds from the exocrine glands (e.g. Bergmann et al. 2004) that elicited significant responses in the male's antenna (Löfstedt et al. 1994, 2008; Jewett et al. 1996; Bjostad et al. 1996; Larsson and Hansson 1998; Bergmann et al. 2001). However, in some cases, females also respond to the active compounds (e.g. Jewett et al. 1996) and males also produce the active compounds that elicit the electroantennographic response in the male antenna (*e.g.* Bergmann et al. 2001). All together, these data point towards a less specific role of the Trichoptera exocrine glands when compared to Lepidoptera (Löfstedt et al. 1994). In Lepidoptera, males are usually the ones adapted to sense very small amounts of female pheromone compounds and thus, sexual communication is more specific.

#### Conclusion

While trichopteran larvae have very small antennae with only one type of non-chemical sensilla, adults have well developed antennae with a wide array of sensilla. In addition, several researchers have been investigating chemodetection in caterpillars and its contrast to the adult counterpart. Thus, it would be of interest to compare the extensive findings that have already been published on several lepidopterans with those of trichopterans. For example, does the fact that the larva is anosmic reflect changes in the brain structure and physiology of the adult stage? If so, how do these changes compare with those of lepidopterans? These questions can shed light on the encoding of chemosensory modalities and, thus, on the behavioral repertoires that these animals exhibit in their sexual communication.

### Diptera

In contrast to the orders discussed before, the order Diptera has been extensively researched and mosquitoes, due to their role as vectors of human diseases have been particularly well examined (Clements 1999). The following section is simply a summary of some examples (mainly mosquitoes) that are relevant for this review. In contrast to adults, larvae have been the focus of fewer studies and for this reason more in-depth information is provided on the larval instar.

#### Antennal morphology and type of sensilla Larvae

The order Diptera is comprised of the suborders Nematocera (thread-horned flies) and Brachycera (short-horned flies). Of the nematoceran families with at least some aquatic larvae only the following have had their antennal sensilla described so far: Culicidae (mosquitoes), Simuliidae (blackflies), Chaoboridae (phantom midges), and Psychodidae (drainflies). The speciose Chironomidae (non-biting midges) and Ceratopogonidae (biting midges) have not been described so far. Although variation in antennae structure and sensilla occurs in these families, basic characteristics can be recognized (e.g. the presence of a cone organ in almost all the families). The general structure of the reduced antenna of the mosquito larva consists of a ring-like scape, fused pedicel and flagellum. In Culicidae, the antennae of *Aedes aegypti* (Zacharuk et al. 1971; McIver 1982; Gaino and Rebora 1999) and *Toxorhynchites brevipalpis* (Jez and McIver 1980; McIver 1982; Gaino and Rebora 1999) have been extensively studied. In these species, the antenna consists of a single tubular piece or a cylinder ending in a terminal membranous region where the rest of the sensilla are situated. Six types (10 in total) and five types (8 in total) of sensilla are present in *Ae. aegypti* and *T. brevipalpis* respectively; and in both species, a cone organ and a basiconic peg sensillum have been described. In Simulidae, the two-segment antenna is tubular with a membranous base that possesses a bacteria covered multiporous sensillum (Craig and Batz 1982; Gaino and Rebora 1999). Among the five types of sensilla that are present, the chemosensory cone sensillum is worth noting. In *Chaoborus crystallinus* (Chaoboridae), the highly modified prehensile antenna articulates on the anterior tip of the rostrum and consists of seven types of sensilla (Nicastro et al. 1995; Gaino and Rebora 1999). Lastly, *Psychoda cinerea* (Psychodidae) has multimodal receptor fields on the anterior part of the head and each one of these contains eight morphologically different types of sensilla (Gaino and Rebora 1999). More detail on the different types of sensilla is given in Appendix 5.

#### Adults

Although there are reports on the morphology and distribution of sensory receptors on the antenna of non-mosquito nematocerans (e.g. Cribb 1997; Felippe-Bauer and Bauer 1990), research has been strongly biased towards Culicidae. The structure and ultrastructure of the sensilla in the antennae of mosquitoes, as well as their electrophysiological properties, have been extensively studied and compiled elsewhere (e.g. McIver 1982; Sutcliffe 1994; Clements 1999). Due to their importance as disease vectors that afflict human beings, the chemosensitive sensilla in the antenna of female *Ae. aegypti* (e.g. Ghaninia et al. 2007; Ghaninia et al. 2008), *Anopheles gambiae* (e.g. Qiu *et al.* 2006) and *Culex quinquefasciatus* (e.g. Hill et al. 2009), as well as the odor ligands of the olfactory receptor neurons (ORNs) housed in them, have been particularly examined. Appendix 5 summarizes the adult sensilla of mosquitoes and their sensory modalities, but the reader is encouraged to go to the references mentioned in this summary (and the references therein) for a more detailed description on the topic.

#### Brain morphology

Mosquitoes, in particular *Ae. aegypti* and *An. gambiae,* have been the focus of studies on brain morphology and physiology within the Nematocera. Although limited information is available on the olfaction of the larvae (see below), extensive research has been conducted on the adult stage. However, only a summary of the adult's olfactory processes is provided.

While in *Ae. aegypti,* 49 male antennal glomeruli and 50 female antennal glomeruli have been described (fewer glomeruli were reported by Anton 1996) in the antennal lobe, in *An. gambiae* 61 are present in males and 60 in females. A detailed description on the neuronal architecture of the mosquito deutocerebrum (including the antennal lobe and the antennal mechanosensory and motor center) along with a partial functional map of the antennal lobe of the female mosquito is found in Ghaninia (2007). After the publication of the genome of *An. gambiae* (Holt et al. 2002) and *Ae. aegypti* (Nene et al. 2007), the identification of candidate odorant receptors (ORs) in these two species (as well as in *Cu. quinquefasciatus*) has been undertaken by several research groups. In *An. gambiae,* 79 candidate odorant receptors (ORs) and 76 candidate gustatory receptors were identified by Hill et al. 2002. Among these ORs, AgOr7 is a highly conserved receptor gene that is expressed in the majority of the ORNs, as is Dor83b in *Drosophila melanogaster*, and is supposed to be necessary for the functioning of other ORs expressed in ORNs (Pitts et al. 2004). After AgOr7 was characterized, orthologs in *Ae. aegypti* (i.e. AaOr7; Melo et al. 2004) and *Cu. quinquefasciatus* (i.e. CqOr7; Xia and Zwiebel 2006) were also identified. Moreover, the recent study of the ORs in adult *An. gambiae* has shed light on the specific responses of these AgOrs to biologically relevant odors (Wang et al. 2010; Carey et al. 2010) and also allowed for a comparison to *D. melanogaster* taking into account their different ecological needs (Carey et al. 2010). However, after the identification of pheromone binding proteins in moths (Vogt and Riddiford 1981), researchers realized that these proteins (more generally known as odor binding proteins or OBP's) bind and carry odor molecules to the ORs. Today, several OBPs have been identified in several species of mosquitoes (e.g. Ishida et al. 2002; Xu et al. 2003; Sengul and Tu 2008, 2010; Pelletier and Leal 2009) and in at least one case, the ligand established (Biessmann et al. 2010). It is interesting that even though the need for odorant transport is presumably unique to terrestrial animals (Vogt et al. 1991), OBPs have been found in *Ae. aegypti* (Biron et al. 2005), *An. gambiae* (Xu et al. 2003) and *Anopheles stephensi* (Sengul and Tu 2010) larvae.

The selective expression of AgOr7 in a particular tissue is indirect evidence of its chemosensory function that has been used to show that trichoid sensilla in the adult antenna and the distal part of the antennae in the larva are indeed chemosensory structures (Pitts et al. 2004). Interestingly, of the 23 ORs found to be expressed in the larvae of *Ae. aegypti,* eight are also expressed in the antenna of males and females (Bohbot et al. 2007). Xia et al. (2008) found that 12 ORNs labeled with AgOr7 antibody project into the sensory cone of the larva of *An. gambiae* and of 33 odorants tested, larvae responded to 11 (10 of which are aromatics and some products of organic decay). Moreover, some of these compounds (e.g. indole and 4-methylcyclohexanol) have also evoked strong electrophysiological response in the antenna of the female (Blackwell and Johnson 2000). Thus, as suggested by Xia et al. (2008), these compounds might be involved in larval development (by means of food detection) in addition to oviposition site selection in adults.

#### Behavior

##### Larvae

Nematocera species feed on an extremely broad range of plant, animal, and detrital material (Teskey 1984). Information on three families, specifically Chaoboridae, Chironomidae, and Culicidae is available regarding the sensory modalities involved in food searching and predator avoidance. *Chaoborus* has been reported to be predaceous and to probably use mechanical stimuli to detect its prey. Winner and Greber (1980) concluded that *Chaoborus punctipennis* most probably discriminates swimming prey items by detecting differences in water vibrations. In addition, experiments with a vibrating rod or probe determined the preferred distance at which a midge would attack and which frequencies and intensities are more likely to elicit such an action (Guiguère and Dill 1979). Since these experiments were done in the dark and the probe had no odor, the results point towards a mechanically-mediated hunting behavior. However, as in other immature insect stages, Chaoborids may change their behavior in presence of fish (Luecke 1986) and this behavior seems to involve fish kairomones. Chemicals produced by the three-spined stickleback *Gasterosteus aculeatus* affected the vertical migration of *Chaoborus flavicans* and this fish effect persisted for more than 15 days (Dawidowicz et al. 1990). In another experiment with *C. flavicans,* an increase in the tube depth (where these insects retreat) was observed when fish odor was added, but only in larvae that came from a fish lake (as opposed to those from fishless lakes); and the effects faded after some time suggesting microbial degradation of the chemical cues (Oda and Hanzato 2008).

Two reports have documented chemosensation in chironomid larvae. The first one showed that digging, burrowing, and foraging behavior changed after fish-conditioned water was added to a larval tank (Holker and Stief 2005). In these experiments, the response was seen 120 min after exposure and the chironomids were able to assess different infochemical concentrations which are probably indicative of different fish densities. The second set of experiments reported the first insect pheromone acting in an aquatic environment. Naik et al. (2006) demonstrated that the larval cuticle of the non-biting midge *Chironomus ramosus* contained farnesol (among other compounds) and that this “pheromone-like” compound had attractive properties for the larvae implying that farnesol is an aggregation pheromone.

Finally, included in the Culicidae family, several species of some subfamilies have been investigated (see some examples below). Within Toxorhynchitinae, *Toxorhynchites amboinensis* (predator of *Ae. aegypti* and others), have been shown to be attracted to water in which prey have been reared and also to the movement of living prey (Barber and Hirsch 1984). Using a vibrating probe, McIver and Beech (1986) observed that the 3^rd^ instar larvae of *T. brevipalpis* attacked the probe in response to vibrations alone. These authors discarded vision as a factor because the compound eyes are nonfunctional at this stage (Sato 1961 in McIver and Beech 1986) and also chemical cues were discarded due to the fact that the probe is not associated with any prey odors. However, instead of proposing the antennae to be involved in the detection mechanism, they proposed that the main setae in the thorax and abdomen might be responsible for prey sensing, and Magnuson and Baerwald (1987) suggested that the hair sensilla on the head detected prey movement. Within Culicinae, one example of a detritivorous larva attracted to chemical cues from mucilaginous seeds was reported for *Culex pipiens quinquefasciatus* (Page and Barber 1975). Surprisingly predator avoidance has not received the same attention as in other aquatic insects, but the above evidence suggests that, as other aquatic insects, these have evolved to respond to a diverse range of stimulants.

#### Adults

Since the biology of the adult stage of mosquitoes is well studied (e.g. McIver 1982; Clements 1999), only a brief summary based on a review of mosquito sensory responses written by Clements (1999) supplemented with later citations is given.

Male and female mosquitoes need three kinds of resources, namely sugar from plants, mates, and resting sites; in addition, females need blood meals as a protein source and oviposition places. First, experiments done with *Anopheles arabiensis* and *Ae. aegypti* showed that mosquitoes could detect floral odors (Clements 1999) and green leaf volatiles (reviewed by Takken and Knols 1999) and also that these insects fly upwind towards the source (Clements 1999). Second, mating attraction at long distance has been shown to occur in response to sound stimulus, i.e. the male responds to the wing beat frequency of the female (e.g. Warren et al. 2009), but there is also evidence for a contact sex pheromone emanating from the legs of the female *Culiseta inornata* (Lang and Foster 1976 in Clements 1999; Lang 1977 in Clements 1999). Third, regarding host searching in females, chemical cues that have been shown to elicit attraction are: 1) expired breath (CO2 and water vapor); 2) substances secreted by the eccrine (sweat), apocrine (protein, carbohydrates and ammonia), and sebaceous (sebum) glands; 3) epidermal secretions and their bacterial decomposition products; 4) flatus; and 5) urinary and faecal associated contaminants and their bacterial decomposition product (Clements 1999). Besides this, aggregational pheromones released during feeding have also been suggested (e.g. in the sandfly *Phlebotomus papatasi*) to be involved along with other cues (e.g. Kennedy 1938). Lastly, oviposition preference in water where larvae occur (Ikeshoji 1966b in Clements 1999; Rejmánková et al. 2005) and electrophysiological responses in the female antenna when tested with larvae-water chemo-attractants have been confirmed (Blackwell and Johnson 2000). The presence of fish or tadpoles can also affect oviposition preference in gravid females (e.g. Petranka and Fakhoury 1991). These authors suggested that *Anopheles* and *Chaoborus* (phantom midge) females might chemically sample water with their tarsi while they are about to lay eggs. Focks and Hall (1977) found that female *Toxorhynchites rutilus rutilus* (predator of the larvae of *Ae. aegypti*) preferred to oviposit in water previously used to rear *Ae. aegypti.* There is also evidence for an oviposition aggregational pheromone for *Cu. quinquefasciatus* and *Culex tarsalis* used as an indicator of where egg rafts have already been laid (Clements 1999). Apparently, gravid females of these two species respond to a chemical that is released from droplets that become visible at the apices of the eggs soon after they have been laid. Since mosquito oviposition pheromones could be used to lure gravid females, much research has been focused on identifying these odors (e.g. Millar *et al.* 1992; Du and Millar 1999; Olagbemiro et al. 2004; Lindh et al. 2008) and on elucidating how mosquitoes detect them (e.g. Leal et al. 2008; Pelletier et al. 2010a, 2010b). All these data illustrate that, at least in Nematocera (but probably in many other insect orders), adults have evolved to search for optimal environmental conditions in which their offspring would be most likely to survive.

#### Conclusion

Mosquitoes have been extensively studied and present a unique opportunity to understand the connection between the aquatic larval stage and the terrestrial adult. Because both adults and larvae sense and use chemical cues (including some of the same chemistries) and the molecular biology has been worked out, mosquitoes are a good model to study changes in the olfactory system between an aquatic larva and its terrestrial adult. Although *D. melanogaster* is currently the best-studied insect, this model insect is not suited to answer questions about the sensory adaptations to aquatic and terrestrial environments. Recent data on the ORs and OBPs expressed in larvae and adult mosquitoes, together with the finding that some of these are shared by immature and mature stages, will be useful in answering the question of how adults may utilize information that is relevant during the larval stages. Furthermore, the fact that some chemical compounds were behaviorally important in the larva and adult, gives support to the idea that female mosquitoes may be sensing the water before ovipositing and by this ensuring a better environment for their brood (e.g. where more food or less natural predators exist). It would be interesting to see if females of other aquatic insect orders exhibit a similar behavior.

## Discussion

On the one hand, chemosensation has been extensively studied in terrestrial adult insects (e.g. dipterans, lepidopterans, etc.) in terms of the external and internal antennal morphology and the organization of the antennal lobes and higher centers of the brain. On the other hand, the role of chemical cues in insects that inhabit an aquatic environment during either the adult stage (e.g. aquatic coleopterans and heteropterans) or the larval stage (e.g. ephemeropterans and plecopterans) has been studied to a much lesser extent. Besides expanding knowledge on the biology of one of the most successful animal classes, the study of sensory sensation in insects is relevant to understand how information is acquired, stored, and used to elicit particular behaviors. Animals perceive a subjective representation of the world as a consequence of a vast array of sensations ultimately resulting in decision-making. Even though these behaviors can be very elaborate, they must be based on the activity of neural circuits. Therefore, studying the animal brain helps researchers comprehend how biological neural networks process sensory information (see Chittka and Brockmann 2005).

The larval sensilla of holometabolous insects are present in the embryo and are typically replaced during metamorphosis by adult-specific sensilla derived from imaginal discs (reviews: Levine et al. 1995; Truman 1996; Tissot and Stocker 2000). In contrast, as exemplified by *Rhodnius prolixus,* every time hemimetabolous insects molt, the cuticular surface increases and new sensilla appear (Wigglesworth 1940 in Keil 1997). This may well be the case for plecopterans also, but the antennae of both ephemeropterans and odonates are reduced in size after the last molt. Since hemimetabolous insects do not undergo complete metamorphosis as holometabolous insects do, modifications in brain structures associated with change of environment, i.e. from water to land, could be first analyzed in these insects.

The divergence in the role of neurons seen in different species can arise from modifications in the connectivity of those neurons (Katz 2007). Neurons, as the basic units of a neural network, can be connected to elicit a particular behavior (e.g. upwind flight of a male moth in response to sex pheromones) or can be plastic in response (e.g. olfactory learning; Bargmann 2006). Since the physiological properties of neuronal dendrites largely depend on their morphology, characteristics such as size, branching patterns, and relative position of an arbor are indicative of the particular role the cell has (Williams and Truman 2005). In holometabolous insects, during metamorphosis, the central nervous system (CNS) undergoes major remodeling which includes: neurons that function in the larval CNS, but die during metamorphosis; larval-and pupal-born neurons that are only functional in the adult CNS; and neurons that function in both the larval and adult system (by dendrite and axon reorganization during metamorphosis; Tissot and Stocker 2000; Truman 1990). Since the physiology and morphology of neural structures in Lepidoptera has been extensively studied, by comparing these with those of Trichoptera, hypotheses about how well neural networks are preserved across phylogenetic orders and the selective pressure that impacts these nervous systems can be addressed. Katz and Harris-Warrick (1999) suggested that neural circuitries might evolve more slowly than behavior does, and thus, natural selection could modify the array of behaviors produced by a circuit by altering its inputs or by altering how it handles those inputs.

Even though, in holometabolous insects, the adult antennal lobe (AL) develops from a different brain area than that of the larval antennal lobe (Jefferis et al. 2004), at least 15 glomeruli of the AL of *D. melanogaster* adults are innervated by remodeled embryonic projection neurons (PNs; Gerber and Stocker 2007). A similar phenomenon was documented for γ neurons of the mushroom bodies (MB; Gerber and Stocker 2007) and a synchronized remodeling between these MB neurons and persistent projection neurons seems to occur (Marin et al. 2005). If these PNs and MB neurons have a similar function in both stages, this could also provide insights into the gathering and integration of chemical information in the insect brain. Moreover, the order Diptera presents a unique opportunity for comparison between mostly terrestrial larval and adult insects (i.e. flies) and dual-lifestyle insects (i.e. mosquitoes) in this regard. The rewiring of the nervous system, like the other alterations that take place during metamorphosis, occurs in an endocrine environment that has rising titers of ecdysone and 20-hydroxyecdysone (Brown et al. 2005) and has been shown to involve intracellular local and global changes of calcium (Williams and Truman 2005). Once the factors that influence the final form of a neuron are elucidated, our knowledge of how morphology and physiology relate to each other will allow us to understand the evolution of the CNS (see for example Stollewerk and Simpson 2005).

Finally, there are several invertebrate and vertebrate animals that are amphibious and are able to sense chemical cues in aquatic and terrestrial environments. For example, in the beetle *Dytiscus marginalis,* the same concentrations of coumarin and synthetic musk elicited a similar response in water and in air (Schaller 1926 in Jensen and Zacharuk 1991) and by recording from single sensilla, Behrend (1971) found similar results (in Jensen and Zacharuk 1991). Furthermore, behavioral studies in *Laccophilus maculosus* showed that in air, these beetles responded to about 10% of the concentration required to produce a similar response in water (Hodgson 1951, 1953). These results led Jensen and Zacharuk (1991) to propose that multiporous sensilla on the antennae of *Graphoderus occidentalis* are probably olfactory, but may also be sensitive to lower concentrations of chemicals in both air and water. This observation in addition to the finding that some OBPs are found in the antenna of the larva and adult stage of mosquitoes (Xu et al. 2003; Biron et al. 2005; Sengul and Tu 2010) might indicate that the sensing of odors in both media is based on similar molecular and physiological processes. Moreover, the nasal cavity of anurans reorganizes during metamorphosis allowing these animals to continue sensing waterborne odorants while also being able to sense airborne odorants (Belanger and Corkum 2009). This is possibly due to the expression of fish-like and mammalian-like receptor genes utilized in selective recognition of water-soluble or airborne odorants, respectively, in two different compartments of the frog's nose (Freitag et al. 1998). It would be of interest to compare the sensory mechanism of amphibious vertebrates with those of invertebrates and establish the morphological properties of sensory organs that allow for the same sensory modality in two different media.

This review has summarized many studies that demonstrate that many aquatic insects are adapted to sense chemical cues from different sources in their environment and to adjust their behaviors in response to specific cues. Chemosensation is a topic of much interest because of its complexity in stimuli composition when compared to other senses. Insects have been used as models to unravel the intricacy of biological neural networks and have proven to be very useful due to their simplified CNS. The physiological and neurological transformations that aquatic insects undergo throughout their lives are so extreme, that they may be a better model to understand how and why neural remodeling occurs. The vast array of molecular and genetic techniques used to study *D. melanogaster* will soon be available for mosquitoes too, and thus, a comparison between these two closely related insects will provide information about the developmental changes required for a dual lifestyle. Furthermore, Ephemeroptera and Odonata, being lineages of basal insect orders and having nymphs with chemosensory capability in contrast to that of the adult, may offer special cases to help us understand the evolution of sensory parts of the brain and the associated behaviors.

## Abbreviations

**AL**,: antennal lobe;
**CNS**,: central nervous system;
**MB**,: mushroom bodies;
**OR**,: odorant receptor;
**ORN**,: olfactory receptor neuron;
**PN**,: projection neuron
